# Pancreatic Polypeptide but Not Other Members of the Neuropeptide Y Family Shows a Moderate Association With Perceived Anxiety in Obese Men

**DOI:** 10.3389/fnhum.2020.578578

**Published:** 2020-10-19

**Authors:** Selina Johanna Schaper, Tobias Hofmann, Ellen Wölk, Elena Weibert, Matthias Rose, Andreas Stengel

**Affiliations:** ^1^Department for Psychosomatic Medicine, Charité Center for Internal Medicine and Dermatology, Charité-Universitätsmedizin, Corporate Member of Freie Universität Berlin, Humboldt-Universität zu Berlin, and Berlin Institute of Health, Berlin, Germany; ^2^Department of Quantitative Health Sciences, University of Massachusetts Medical School, Worcester, MA, United States; ^3^Department of Psychosomatic Medicine and Psychotherapy, Medical University Hospital Tübingen, Tübingen, Germany

**Keywords:** brain-gut axis, depression, eating disorder, gut-brain axis, obesity, psychosomatic, stress, peptide YY

## Abstract

Neuropeptide Y (NPY), peptide tyrosine tyrosine (PYY), and pancreatic polypeptide (PP) are important mediators in the bidirectional communication along the gut-brain-axis. Best known for their role in the regulation of appetite and food intake they are considered to play a crucial role in the development of obesity. Additionally, mounting evidence indicates a regulatory function in anxiety, mood and stress resilience with potential sex differences. In the present study, we examined the associations of NPY, PYY, and PP plasma levels with anxiety, depressiveness and perceived stress in obese patients. We analyzed 144 inpatients (90 female, 54 male, BMI mean: 49.4 kg/m^2^) in a naturalistic treatment setting for obesity and its somatic and mental comorbidities. Fasting blood samples were taken, and patients completed psychometric self-assessment questionnaires (GAD-7, PHQ-9, PSQ-20) within the first week after admission and before discharge. Plasma concentrations of the peptides were measured by ELISA. Women showed significant higher anxiety (GAD-7: 8.13 ± 5.67 vs. 5.93 ± 5.42, *p* = 0.04) and stress scores (PSQ-20: 52.62 ± 23.5 vs. 41.23 ± 22.53, *p* = 0.01) than men. In the longitudinal analysis women with a clinically relevant improvement of anxiety (≥ 5 points on GAD-7, *p* < 0.001) also showed significant improvements in depression (PHQ-9: 38%, *p* = 0.002) and PSQ-20 scores (23%, *p* = 0.005) while anxiety-improved male patients only improved in the subscale *tension* of the PSQ-20 (34%, *p* = 0.02). In men we observed a positive correlation of PP with anxiety scores (GAD-7: *r* = 0.41, *p* = 0.007) and with age (*r* = 0.49, *p* = 0.001) on admission while NPY negatively correlated with age (*r* = -0.38, *p* = 0.01). In contrast, there were no significant associations (*p* > 0.05) in female subjects in the cross-sectional as well as in the longitudinal analysis. In conclusion, women suffering from morbid obesity showed greater psychological comorbidity and considerable interactions among them. Despite that we solely observed associations of PP with anxiety and age with NPY and PP in men, suggesting a possible influence of sex hormones on the NPY system. However, improvement of anxiety scores did not lead to significant changes in NPY.

## Introduction

Obesity, defined by the WHO as a body mass index (BMI) ≥ 30 kg/m^2^ (World Health Organisation [WHO], 2020), is one of the major health problems of the 21st century. In recent years its prevalence has increased dramatically and thus became a global epidemic (NCDRF, 2016). Obesity is often accompanied by other health implications including a number of preventable chronic illnesses such as diabetes, cardiovascular diseases and cancer that are among the leading causes of death (GBD 2015 Obesity Collaborators Afshin et al., 2017). The concept of energy imbalance with excess dietary calories is considered to play a pivotal role in the development of obesity. In modern societies prevails the unrestricted availability of food and, therefore, human food consumption cannot solely be attributed to self-preservation. Emerging evidence suggests a strong impact of hedonic mechanisms in the control of food intake which involves cognitive, reward and emotional processes (Horwath et al., 2020).

Eating behavior is regulated by the gut-brain axis, the bidirectional communication between the central (CNS) and the enteric nervous system. Neural (Strandwitz, 2018), immunological (Fung et al., 2017), endocrine (Kuhne and Stengel, 2019) and gut microbiota-derived (Torres-Fuentes et al., 2017) messengers are closely interrelated in the regulation of appetite and food intake. Neuropeptide Y (NPY), peptide tyrosine tyrosine (PYY) and pancreatic polypeptide (PP) represent a family of peptide hormones consisting of 36 amino acids. In humans, these peptides act with particular affinities *via* four subtypes of G-protein-coupled receptors (Y1, Y2, Y4, Y5) (Pedragosa-Badia et al., 2013) as important mediators along the gut-brain axis. NPY is one of the most potent orexigenic peptides that is abundantly expressed within the central and peripheral nervous system (PNS) (Holzer et al., 2012). Centrally, NPY displays its highest expression within hypothalamic nuclei (Bai et al., 1985), particularly the arcuate nucleus (ARC) that plays a key role in the regulation of hunger and satiety (Myers and Olson, 2012). Within the gastrointestinal tract, the primary source of NPY originates from enteric neurons (Cox, 2007). NPY is known to influence several processes involved in energy homeostasis, including energy intake and expenditure, physical activity and adipose tissue function (Loh et al., 2015). PP and PYY are postprandially released gut-derived peptides that inhibit food intake (Field et al., 2010). PYY_3__–__36_, the biologically active form accountable for its anorexigenic effects, is mainly produced by intestinal L cells, whereas PP is synthesized by endocrine F cells of the pancreatic islets (Ekblad and Sundler, 2002). The release of both peptides occurs proportionally to the preceding caloric intake (Adrian et al., 1976, 1985) and leads to the inhibition of orexigenic pathways in the hypothalamus (Shi et al., 2013). PYY inhibits the release of NPY in the ARC *via* Y2 receptors (Ghamari-Langroudi et al., 2005), while PP preferably binds to Y4 receptors to modulate vagal cholinergic pathways in the brainstem (McTigue et al., 1997) and the expression of several hypothalamic feeding-regulatory peptides (Asakawa et al., 2003; Lin et al., 2009). Reduced circulating PYY (Batterham et al., 2003) and PP (Marco et al., 1980) as well as elevated NPY (Baltazi et al., 2011) levels which can be found in obese subjects give rise to a role in the pathophysiology of obesity.

Besides controlling ingestion and energy homeostasis the NPY family also appears to have an impact on emotional-affective behavior. Chronic psychosocial stress is frequently linked to obesity and metabolic disorders (Scott et al., 2012). Mounting evidence indicates that NPY might promote some of the underlying pathomechanisms. Particularly, stress-induced sympathetic, glucocorticoid and hypothalamic activity leads to upregulated NPY expression which in turn results in increased food intake (Zakrzewska et al., 1999) and growth of abdominal fat (Kuo et al., 2007). In addition, postprandial PYY secretion is inhibited under conditions of psychological stress (Kiessl and Laessle, 2016). Moreover, NPY and its receptors are broadly expressed in brain areas, e.g., amygdala and the prefrontal cortex, that mediate stress resilience (Kask et al., 2002).

Anxiety is a fundamental response to stress. In rodents, NPY exerts anxiolytic effects in the amygdala, primarily *via* Y1 receptor activation (Sajdyk et al., 1999), although other receptors might be involved as well (Fendt et al., 2009). On the contrary, stimulation of the Y2 receptor promotes an anxiogenic response (Sajdyk et al., 2002) presumably due to presynaptic inhibition of NPY release (Caberlotto et al., 2000). In humans it was found that genetic variations with low-expression NPY genotypes display a maladaptive responsiveness to stress that predispose to major depression (Mickey et al., 2011) and anxiety disorders (Amstadter et al., 2010). Animal studies indicate an implication of PP and PYY in mediating regulation of emotion, however, the underlying pathways remain elusive. While the deletion of PYY increases depression-like behavior but does not affect anxiety (Painsipp et al., 2011), peripherally administered PP promotes extinction learning of cued fear by acting on central Y4 receptors in mice (Verma et al., 2016). However, intracerebroventricular (icv) injection of PP does not affect anxiety (Asakawa et al., 1999) and it is unclear whether PP is generally produced in the brain (DiMaggio et al., 1985).

Mood and anxiety disorders are common comorbidities among obese patients (Rajan and Menon, 2017) and their joint occurrence is related to poorer physical and mental quality of life (QoL) (Nigatu et al., 2016). The exact mechanisms of this association still warrant further investigation of the signaling pathways involved. This is crucial to provide a better understanding and potential treatment options. Therefore, in the present study we investigated the relationship between circulating levels of the members of the NPY family (NPY, PYY, PP) and psychometrically evaluated anxiety, depressiveness and perceived stress in obese psychosomatic patients.

## Materials and Methods

### Subjects

Within the course of a consecutive biosampling study 144 obese inpatients (54 male, 90 female) who received medical treatment for obesity-related somatic and mental comorbidities were recruited upon admission to the Department of Psychosomatic Medicine at Charité - Universitätsmedizin Berlin (between September 2010 and December 2015). All patients were at the age of ≥ 18 years and met the criteria for obesity with a body mass index (BMI) ≥ 30 kg/m^2^. Their treatment consisted of biomedical therapy, individually adapted therapeutic exercise and both individual and group psychotherapy as well as music and art therapy. Patients with current pregnancy or malignant disease, psychotic disorders, somatoform disorders of the gastrointestinal system, preceding bariatric surgery, hypercortisolism and untreated hypothyroidism were excluded from the study.

All patients gave written informed consent. Investigations were conducted in accordance with the Declaration of Helsinki; the study was approved by the institutional ethics committee of the Charité - Universitätsmedizin Berlin (protocol number: EA1/114/10).

### Laboratory Analyses

Venous blood samples were taken after an overnight fast between 7:00 and 8:00 AM within the first week after admission (T0) and at a second time point during treatment (Tx). Patients were advised not to smoke or exercise and only to have a small amount of water before blood withdrawal. Circulating glucose levels were determined by photometric measurement from blood collection tubes containing sodium fluoride that were kept at room temperature. Blood for measuring peptide concentrations was collected in pre-cooled standard EDTA tubes prepared with aprotinin (1.2 Trypsin Inhibitory Unit per 1 ml blood; ICN Pharmaceuticals, Costa Mesa, CA, United States) for peptidase inhibition. After blood withdrawal the tubes were stored on ice and centrifuged for 10 min at 3000 rpm at 4°C. Plasma was separated and samples were stored at −80°C until further processing. NPY, PYY and PP levels were determined using a commercial enzyme-linked immunosorbent assay (ELISA, catalog # EK-049-03, EK-059-02 and EK-054-02, Phoenix Pharmaceuticals, Inc., Burlingame, CA, United States). According to the manufacturer’s information there is no cross-reactivity (0%) between the respective peptide antigens. All samples were processed in one batch; the intra-assay variability was 8, 5, and 5%, respectively.

### Anthropometric Measurements

Body weight and height were assessed at the same day of blood withdrawal in patients wearing light underwear and BMI was calculated as kg/m^2^. Medications and existence of comorbidities were recorded at admission and after hospital discharge.

### Psychometric Parameters

For psychometric assessment patients were asked to complete the following questionnaires and results obtained between two days before and five days after the respective blood withdrawals were accepted. Two modules of the self-administered patient health questionnaire (PHQ) (Spitzer et al., 1999) were used for the assessment of anxiety (GAD-7) and depression (PHQ-9).

The generalized anxiety disorder questionnaire (GAD-7) consists of seven items with scores ranging from “0” (not at all) to “3” (nearly every day) with a maximum of 21 points (Spitzer et al., 1999). The GAD-7 is an efficient tool to measure general anxiety disorder and is also suited to detect symptoms of social anxiety, posttraumatic stress and panic disorder (Spitzer et al., 2006). In the present study we used the German version (Lowe et al., 2008) that showed an internal consistency (Cronbach’s α) of 0.90.

The PHQ-9 depression scale is a 9-item screening instrument for the diagnosis of major depression and the evaluation of the severity of depressive symptoms. Total scores range from 0–27, while the nine items represent the DSM-IV diagnostic criteria for depressive disorders (Spitzer et al., 1999). Our patients were handed out the German version by Löwe et al. (2002). Cronbach’s α in the present sample was determined as 0.88. In a meta-analysis of 17 validation studies in different languages including the German language translation, specificity was 0.92 and sensitivity 0.80 for the diagnosis of a major depressive disorder (Gilbody et al., 2007).

Evaluation of perceived stress was conducted by using the revised 20-item German version (PSQ-20) (Fliege et al., 2005) of the perceived stress questionnaire (PSQ; 30 items) (Levenstein et al., 1993). Providing four subscales the PSQ assesses “worries,” “tension,” “joy” as stress responses and “demands” as perception of external stressors and thereby emphasizes the subjective experience of stress. In the present sample Cronbach’s alpha for the subscales ranged from 0.84 to 0.91.

### Statistical Analyses

Data are expressed as mean ± standard deviation (SD). Normal distribution was evaluated by the Kolmogorov-Smirnov test. Differences between groups were calculated using t-tests and chi-square tests. Pearson’s correlation was calculated to assess associations of two variables. Statistical differences and correlations were considered significant when *p* < 0.05. All statistical analyses were conducted using Sigma Stat 3.1 (Systat Software, San Jose, CA, United States).

## Results

### Demographic, Socioeconomic and Medical Characteristics

The mean observation period between first (T0) and second (Tx) blood sample and psychometric assessment was 2.3 ± 1.2 weeks (range: 1–7 weeks). Demographic and socioeconomic characteristics as well as comorbidities and current medications of the study population are presented in Table 1. In the whole study sample mean age was 46.2 ± 13.4 with a range of 19–73 years and mean BMI was 49.1 ± 9.3 kg/m^2^. Men and women did not differ regarding BMI (48.5 ± 9.3 vs. 49.5 ± 9.3 kg/m^2^, *p* = 0.528), age (47.0 ± 14.1 vs. 45.7 ± 13.0 years, *p* = 0.592) and socioeconomic characteristics (*p* > 0.050; Table 1). However, significant sex differences were observed in the prevalence of obesity-related comorbid conditions and medication. More precisely, sleep-associated breathing disorder (*p* = 0.011), hypertriglyceridemia (*p* = 0.007), fatty liver disease (*p* = 0.031) and type 2 diabetes that requires insulin treatment (*p* = 0.014) were more frequent in men. Psychopharmacological medication was given to 33.3% of the study population with selective serotonin reuptake inhibitors (SSRI) and selective serotonin-norepinephrine reuptake inhibitors (SNRI) being the most frequent pharmaceuticals (19.4%).

**TABLE 1:** Demographic and socioeconomic characteristics, comorbidities and medication of study patients.

**Parameter**	**Whole population**	**Women**	**Men**	***P***
	**(*n* = 144)**	**(*n* = 90)**	**(*n* = 54)**	
Age (years)	46.2 ± 13.4	45.7 ± 13.0	47.0 ± 14.1	0.592
BMI (kg/m^2^)	49.1 ± 9.28	49.5 ± 9.28	48.5 ± 9.33	0.528
**Socioeconomic characteristics**				
Living in a partnership	66 (45.8%)	41 (45.6%)	25 (46.3%)	0.931
**Level of education**				0.76
University entrance diploma	27 (18.8%)	13 (14.4%)	14 (25.9%)	
Vocational diploma	9 (6.25%)	6 (6.67%)	3 (5.56%)	0.444
Secondary education certificate	58 (40.3%)	44 (48.9%)	14 (25.9%)	0.897
Basic school qualification	42 (29.2%)	23 (25.6%)	19 (35.2%)	
No school-leaving qualification	8 (5.56%)	4 (4.44%)	4 (7.41%)	
Currently employed	51 (35.4%)	34 (37.8%)	17 (31.5%)	
Unemployment during past 5 years	65 (45.1%)	41 (45.6%)	24 (44.4%)	
**Comorbidities**				
Binge eating disorder	21 (14.6%)	15 (16.7%)	6 (11.1%)	0.334
Sleep-associated breathing disorder	74 (51.4%)	38 (42.2%)	36 (66.7%)	**0.011**
Type 2 diabetes mellitus	49 (34.0%)	27 (30.0%)	22 (40.7%)	0.188
Arterial hypertension	96 (66.7%)	56 (62.2%)	40 (74.1%)	0.144
Hypercholesterinemia	87 (60.4%)	54 (60.0%)	33 (61.1%)	0.895
Hypertriglyceridemia	40 (27.8%)	18 (20.0%)	22 (40.7%)	**0.007**
Hyperuricemia	65 (45.1%)	37 (41.1%)	28 (51.9%)	0.21
Fatty liver disease	91 (63.2%)	53 (58.9%)	38 (70.4%)	**0.031**
**Medication**				
Insulin	15 (10.4%)	5 (5.56%)	10 (15.1%)	**0.014**
DPP4 inhibitors/GLP-1 analog	7 (4.86%)	3 (3.33%)	4 (7.41%)	0.271
Antidiabetics (other)	26 (18.1%)	16 (17.8%)	10 (18.5%)	0.911
Psychopharmacological treatment	48 (33.3%)	34 (37.8%)	14 (25.9%)	0.144
Neuroleptics	17 (11.8%)	11 (12.2%)	6 (11.1%)	0.841
SSRI/SNRI	28 (19.4%)	21 (23.3%)	7 (13.0%)	0.128
Tricyclic antidepressants	12 (8.33%)	10 (11.1%)	2 (3.70%)	0.12
Other antidepressants	10 (6.94%)	7 (7.78%)	3 (5.56%)	0.612
Tranquilizers, sedatives, hypnotics	3 (2.08%)	2 (2.22%)	1 (1.85%)	0.88
Other psychopharmacological medication	7 (4.86%)	4 (4.44%)	3 (5.56%)	0.764

*Statistical analysis: Data are expressed as mean ± standard deviation. Differences between two groups were assessed using the t-test or chi-square test. Significant differences are displayed in bold. BMI, body mass index; DPP4, dipeptidyl peptidase-4; GLP-1, glucagon-like peptide-1; SNRI, serotonin-norepinephrine reuptake inhibitor; SSRI, selective serotonin reuptake inhibitor.*

### Baseline Patient-Reported Outcomes

In the cross-sectional analysis women showed significant higher levels of anxiety (8.13 ± 5.67 vs. 5.93 ± 5.42, *p* = 0.038) and perceived stress total scores (52.62 ± 23.5 vs. 41.23 ± 22.53, *p* = 0.010) than men (Table 2). This also applied for the PSQ-20 subscales “worries” (*p* = 0.005), “tension” (*p* = 0.015) and “demands” (*p* = 0.035) but not for the subscale “joy” (*p* = 0.110), while all stress subscales highly correlated with each another in both sexes (*p* < 0.001).

**TABLE 2:** Endocrine and psychometric parameters in the cross-sectional analysis according to sex.

**Parameter**	**Whole population**	**Women**	**Men**	***P***
	**(*n*=122)**	**(*n*=77)**	**(*n*=45)**	
NPY (ng/ml)	1.31 ± 0.56	1.33 ± 0.57	1.29 ± 0.57	0.701
PP (ng/ml)	1.66 ± 0.72	1.66 ± 0.68	1.67 ± 0.78	0.929
PYY (ng/ml)	1.28 ± 0.40	1.31 ± 0.41	1.22 ± 0.37	0.244
Fasting glucose (mg/dl) (women: *n* = 68; men: *n* = 42)	113.8 ± 40.5	108.8 ± 35.7	121.8 ± 46.7	0.104
GAD-7	7.32 ± 5.66	8.13 ± 5.67	5.93 ± 5.42	**0.038**
PSQ-20 total	48.4 ± 23.7	52.6 ± 23.5	41.2 ± 22.5	**0.01**
- Worries	46.5 ± 28.6	52.0 ± 28.4	37.2 ± 26.7	**0.005**
- Tension	50.9 ± 28.1	55.6 ± 27.7	42.8 ± 27.3	**0.015**
- Joy	44.8 ± 26.5	41.8 ± 27.0	49.8 ± 25.3	0.11
- Demands	41.0 ± 23.7	44.8 ± 23.5	34.7 ± 24.6	**0.035**

*Statistical analysis: Data are expressed as mean ± standard deviation. Differences between two groups were assessed using the t-test. Significant differences are displayed in bold. BMI, body mass index; GAD-7, Generalized Anxiety Disorder questionnaire; NPY, neuropeptide Y; PHQ-9, Patient Health Questionnaire; PP, pancreatic polypeptide; PSQ-20, Perceived Stress Questionnaire; PYY peptide YY_(3__–__36)_.*

### Associations of Peptides With Age and Anxiety

**FIGURE 1:**
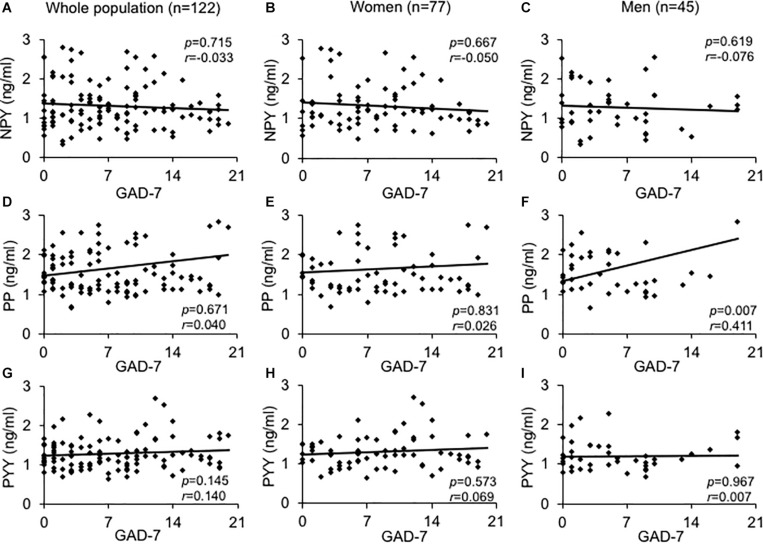
Association between GAD-7 scores and baseline plasma levels of the neuropeptide Y family in obese psychosomatic inpatients. Correlations were assessed between plasma neuropeptide Y **(A–C)**, pancreatic polypeptide **(D–F)**, and peptide YY **(G–I)** in the obese population of both sexes **(A,D,G)** and separately for obese women **(B,E,H)** and men **(C,F,I)**. Values for *r* and *p* are indicated in the figure.

We observed a positive correlation between PP and PYY in men (*r* = 0.430, *p* = 0.004) and women (*r* = 0.843, *p* < 0.001) while NPY and PYY (*r* = 0.336, *p* < 0.030) were associated solely in the male study population. On admission, in men age was negatively associated with NPY (*r* = -0.378, *p* = 0.011) and positively correlated with PP (*r* = 0.492, *p* < 0.001; data not shown). Male subjects also displayed a positive correlation of PP with GAD-7 scores (*r* = 0.411, *p* < 0.007), while NPY and PYY did not (Figure 1). In women neither NPY, PYY nor PP correlated with GAD-7 scores (Figure 1).

### Potential Confounders

Fasting blood glucose did not significantly differ between male and female patients (*p* = 0.200). Moreover, we did not find any correlations between peptides and blood glucose levels in male (NPY: *r* = -0.014, *p* = 0.375; PYY: *r* = 0.064, *p* = 0.688; PP: *r* = 0.131, *p* = 0.408) nor female subjects (NPY: *r* = 0.031, *p* = 0.804; PYY: *r* = 0.149, *p* = 0.225; PP: *r* = 0.182, *p* = 0.137). In the whole sample 7 (4.68%) patients were taking dipeptidyl peptidase-4 (DPP4) antagonists as treatment for type 2 diabetes (Table 1). Male subjects taking DPP4-inhibitors displayed significantly lower NPY plasma concentrations (*p* = 0.018) compared to patients without. After exclusion of subjects taking DPP4-inhibitors the association of PP and anxiety in the male subgroup remained significant (*r* = 0.427, *p* = 0.008).

### Psychometric Parameters and Peptides According to Changes of Anxiety

In the longitudinal analysis patients with a clinically relevant improvement of anxiety (≥ 5 points on GAD-7, *p* < 0.001) displayed significantly higher basal anxiety levels (*p* < 0.001) as well as higher PHQ-9 (*p* < 0.001) and PSQ-20 scores (*p* < 0.001) than patients with no change (± 1 point) or worsening of anxiety (≥ 5 points on GAD-7). Regarding baseline peptide levels, no significant differences in PP (*p* = 0.47) and PYY (*p* = 0.09) were observed in subjects with improvement *vs*. no improvement of anxiety.

### Course of Psychometric Parameters, NPY and BMI During Treatment

Over the observation period, women with an improvement of anxiety also showed significant improvements in PHQ-9 (36%, *p* = 0.002) and PSQ-20 subscales “worries” (30%, *p* < 0.002), “tension” (27%, *p* = 0.003) as well as the total stress score (23%, *p* = 0.005), while “demands” showed a trend toward an improvement” (25%, *p* = 0.052; Table 3). Male patients only improved in the subscale “tension” (34%, *p* = 0.024; Table 4). In patients who did not show relevant alterations in anxiety scores (± 1 point on GAD-7) no significant changes in other psychometric measurements (*p* > 0.050) were observed. BMI did not significantly change during the observation period in women or men with or without an improvement of anxiety scores. Lastly, improvement of anxiety scores did not lead to significant changes in plasma NPY levels (*p* > 0.050).

**TABLE 3:** BMI, psychometric and endocrine parameters in the course of treatment according to the changes of anxiety in women.

	**Before treatment**	**After treatment**	**% change**	***p***
**No change of anxiety** (± 1 point, *n* = 20)				
GAD-7 score	6.85 ± 6.14	6.65 ± 6.12	−2.92	0.918
PHQ-9 score	8.56 ± 6.97	7.75 ± 7.03	−9.46	0.725
PSQ-20 score	50.8 ± 24.7	47.3 ± 22.5	−6.69	0.634
- Worries	47.7 ± 26.4	39.7 ± 29.9	−16.8	0.371
- Tension	52.7 ± 28.2	49.0 ± 26.9	−7.02	0.676
- Joy	47.0 ± 29.2	45.0 ± 23.9	−4.26	0.814
- Demands	50.0 ± 24.4	45.3 ± 22.8	−9.4	0.536
NPY (ng/ml)	1.11 ± 0.53	0.98 ± 0.45	−11.7	0.41
BMI (kg/m^2^)	48.7 ± 8.57	47.8 ± 8.33	−1.85	0.744
**Improvement of anxiety** (≥ 5 points, *n* = 25)				
GAD-7 score	13.4 ± 4.46	5.88 ± 3.72	−56	**<0.001**
PHQ-9 score	13.4 ± 5.01	8.65 ± 4.99	−35.5	**0.002**
PSQ-20 score	68.1 ± 19.2	52.3 ± 18.6	−23.1	**0.005**
- Worries	72.8 ± 25.1	50.9 ± 19.8	−30	**0.001**
- Tension	73.9 ± 21.1	53.6 ± 24.7	−27.4	**0.003**
- Joy	30.4 ± 24.4	37.1 ± 20.3	+ 21.9	0.299
- Demands	56.0 ± 26.7	41.9 ± 23.5	−25.3	0.052
NPY (ng/ml)	1.15 ± 0.44	1.04 ± 0.35	−9.79	0.32
BMI (kg/m^2^)	49.5 ± 9.51	48.5 ± 8.99	−2.12	0.691

*It is to note that three patients showed a worsening of their anxiety (≥ 5 points); however, the n of this group was too small in order to perform meaningful comparisons; therefore these data are not shown in this table. Statistical analysis: Data are expressed as mean ± standard deviation. Differences between two measurements were assessed using the t-test. Significant differences are displayed in bold. BMI, body mass index; GAD-7, Generalized Anxiety Disorder questionnaire; NPY, neuropeptide Y; PHQ-9, Patient Health Questionnaire; PSQ-20, Perceived Stress Questionnaire.*

**TABLE 4:** BMI, psychometric and endocrine parameters in the course of treatment according to the changes of anxiety in men.

	**Before treatment**	**After treatment**	**% change**	***p***
**No change of anxiety** (± 1 point, *n* = 17)				
GAD-7 score	3.5 ± 3.30	3.00 ± 3.34	−13.6	0.676
PHQ-9 score	5.56 ± 5.01	4.35 ± 4.55	−21.8	0.451
PSQ-20 score	34.8 ± 20.9	31.9 ± 20.2	−8.46	0.687
-Worries	31.0 ± 22.5	25.5 ± 20.4	−17.7	0.414
-Tension	36.5 ± 25.5	31.0 ± 24.0	−15.1	0.553
-Joy	57.3 ± 24.4	58.8 ± 27.5	+ 2.74	0.861
-Demands	29.0 ± 22.5	29.8 ± 21.6	+ 2.70	0.877
NPY (ng/ml)	0.94 ± 0.41	0.83 ± 0.33	−11.1	0.425
BMI (kg/m^2^)	48.1 ± 7.01	46.9 ± 6.59	−2.65	0.588
**Improvement of anxiety** (≥ 5 points, *n* = 12)				
GAD-7 score	10.8 ± 3.91	3.33 ± 2.93	−69	**<0.001**
PHQ-9 score	11.4 ± 6.92	7.40 ± 5.60	−35.2	0.156
PSQ-20 score	59.7 ± 24.8	43.5 ± 23.8	−27.2	0.116
- Worries	55.6 ± 30.4	34.4 ± 28.6	−38	0.093
- Tension	69.4 ± 23.9	45.6 ± 24.4	−34.4	**0.024**
- Joy	33.3 ± 20.9	41.1 ± 27.8	+23.3	0.447
- Demands	47.2 ± 34.9	35.0 ± 24.0	−25.9	0.328
NPY (ng/ml)	0.94 ± 0.36	0.74 ± 0.22	−21.4	0.114
BMI (kg/m^2^)	50.8 ± 11.4	49.5 ± 11.3	−2.54	0.783

*Statistical analysis: Data are expressed as mean ± standard deviation. Differences between two measurements were assessed using the t-test. Significant differences are displayed in bold. BMI, body mass index; GAD-7, Generalized Anxiety Disorder questionnaire; NPY, neuropeptide Y; PHQ-9, Patient Health Questionnaire; PSQ-20, Perceived Stress Questionnaire.*

## Discussion

While the three members of the NPY family are commonly known as potent hunger and satiety signals (Chee and Colmers, 2008; Field et al., 2010; Loh et al., 2015) their involvement in emotion regulatory processes has emerged as well. The aim of the presented study was to examine the association of circulating NPY, PYY, and PP with self-reported symptoms of anxiety, perceived stress and depression and in obese individuals as well as their alterations depending on the course of psychometric measures of anxiety during inpatient treatment.

We found higher symptom severity of anxiety and stress in female subjects compared to men, a finding in line with our previous studies (Hofmann et al., 2015, 2017) while another study found an increase in BMI among overweight women with high anxiety symptomatology in GAD-7 but not among men (Demmer et al., 2015). Additionally, women who achieved an improvement of anxiety scores also showed a significant reduction of depressiveness and perceived stress during treatment. These findings likely reflect the generally higher prevalence of depressive and anxiety disorders amongst women (Gater et al., 1998) as well as the frequent co-occurrence and mutual interference of these symptoms (Gros et al., 2013). Initial anxiety scores had to be high enough to enable their significant improvement in the first place. Consequently, patients with a clinically relevant improvement of anxiety presented with higher baseline levels of anxiety, depressiveness and perceived stress. These differences were not reflected in basal peptide concentrations.

The main finding of our study was a moderate positive correlation of PP and anxiety scores measured by GAD-7 which was solely observed in male subjects. The low strength of the relationship could be due to the small sample size of the male subgroup. In this context it is further worth mentioning that the male study cohort was more strongly affected by obesity-related diseases, particularly insulin dependency in type 2 diabetes (see Table 1). Since PP has been shown to mediate glucose homeostasis (Seymour et al., 1996) and circulating peptide concentrations are altered in type 2 diabetic patients (Floyd et al., 1976) we took fasting blood sugar levels as a confounding variable into consideration. However, we did not find any correlations between fasting blood glucose levels and PP or the other members of the NPY family. As mentioned in the introduction, Verma et al. (2016) described that peripheral injection of a selective Y4 agonist facilitates extinction learning of cued fear which could be interpreted as a contradiction to our own observations. On the other hand, PP did not affect fear acquisition and consolidation. A conceivable explanatory approach reconciling these findings with our own results could be that PP secretion is upregulated under the state of anxiety. From an evolutionary point of view anorexigenic effects of PP could benefit reduced risk-taking behavior in face of an acute threat. However, self-limitation of the underlying mechanisms would appear as important to assure long-term survival which could explain why PP simultaneously promotes extinction of conditioned fear but only in fasted and not in fed mice. Both findings reflect the hypothesis that PP is able to mediate anxiety-like behavior through mechanisms from the periphery since Y4 receptors are widely expressed in areas within reach of the peripheral blood circulation and its peripheral administration leads to an activation of regions critical to emotion regulation.

Laboratory analysis showed a negative correlation of age with NPY and a positive correlation with PP in men. Current literature suggests alterations in NPY system function with age, yet its precise role has not been completely clarified. Studies in rodents found age-related changes of NPY concentrations in brain and peripheral tissues, specifically an age-induced reduction in hypothalamic NPY (Pavia and Morris, 1994). Moreover, icv administered NPY stimulated food intake in young rats, while the effect diminished with increasing age up to its absence in old rats (Akimoto and Miyasaka, 2010). However, the attenuated responsiveness to NPY cannot be attributed to a decrease in number and expression of hypothalamic Y1 or Y5 receptors which are thought to mediate the orexigenic effects (Duhault et al., 2000) since older rats display lower Y1 and Y5 mRNA levels but an increased number of neurons transcribing Y1. This might be due to a compensatory mechanism (Coppola et al., 2004). In humans, NPY levels in the cerebrospinal fluid of women, but not men, increased significantly with aging (Taniguchi et al., 1994). It should be noted that in the present study NPY was measured peripherally which does not necessarily reflect its central expression. Nevertheless, consistent with our results a prior study investigating male subjects also demonstrated a decline of plasma NPY levels with increasing age as well as no correlation with BMI (Chiodera et al., 2000). Lastly, our observations match previous findings that found lower plasma PP in adults compared to children (Hanukoglu et al., 1990).

Contrary to our expectations, we did not find a significant association of NPY with depressiveness. In this case our results share a number of similarities with one previous study. Using the same tool for assessing depression (PHQ-9), Zheng et al. (2016) also did not detect a significant correlation with circulating NPY. However, these authors observed a positive association of DPP4 and depressiveness alongside an inverse correlation of NPY with increased DPP4 activity indicating their possible interaction in the pathogenesis of depression. DPP4-mediated proteolytic degradation of NPY and PYY results in altered signaling and functionality of respective peptides (Elmansi et al., 2019). A considerable proportion of our study population (34%) suffered from type 2 diabetes, a condition in which plasma DPP4 activity is altered (Mannucci et al., 2005; McKillop et al., 2008) and DPP4 inhibitors are commonly used oral antidiabetics. Male subjects taking DPP4 inhibitors displayed significantly decreased NPY levels (*p* = 0.02) compared with the remaining population while all psychometric parameters and other peptide values were not affected. After excluding subjects taking DPP4 inhibitors association of PP and anxiety remained significant which concurs well with previous results that found PP secretion to be unaffected by DPP4 inhibition (Veedfald et al., 2015). However, DPP4 activity correlates with various parameters which are altered within the course of metabolic syndrome (Lamers et al., 2011). Therefore, we cannot exclude that lacking correlations between other peptides and psychometric measurements were caused by varying manifestations and severity of obesity-related disorders in the study population.

Another investigation indicated decreased NPY plasma concentrations in patients with major depressive disorder compared to healthy controls (Hashimoto et al., 1996). Incongruous evidence might be attributed to the co-occurrence of obesity and depression that are opposingly interrelated with NPY and conceivably offset one another. Indeed, the former study observed a positive association between NPY and BMI (Zheng et al., 2016) which has not been observed here, which might be due to antidepressants and concomitant diseases acting as confounding factors. Particularly, SSRI and SNRI which constituted the most frequent psychopharmacological medications taken by our study participants have been shown to interact with NPY (Lee et al., 2016).

NPY is known to be involved in the stress reaction (Reichmann and Holzer, 2016). Even though we did not find a significant correlation between NPY and perceived stress in our study population, a statistically significant negative association with the PSQ subscale “joy” was only narrowly missed in men. According to its validation study the subscale joy reflects individual resources and is positively correlated with QoL (Fliege et al., 2005). Thus, NPY may play a role in mediating stress resilience, a hypothesis to be further investigated.

Remarkably, female subjects did not show any correlation between peptides and other variables in the present study. Observed sex differences could be explained by disparities in hormonal status. Specifically, estradiol exhibits an inhibitory effect on NPY expression in the ARC of female rats (Reboucas et al., 2016), while increased NPY levels under estrogen deficiency, for instance during menopause, led to hyperphagia and body weight gain (Ainslie et al., 2001). In order to eliminate the impact of medical treatment as a potentially confounding factor in the cross-sectional analysis sample collection was carried out at the beginning of the hospital stay. Thus, an adjustment for menstrual cycle phase in female subjects was not feasible.

Contrary to our hypothesis, improvement of anxiety scores did not lead to significant changes in circulating NPY levels which was the main focus of this study. Other members of the NPY family were not determined in the longitudinal analysis which should be further investigated in the future with respect to the observed association of PP with anxiety scores in men.

Further limitations of the present study are noteworthy. First, all psychometric measurements used self-assessment questionnaires. Despite the substantial advantages of reflecting the subjectively perceived state of emotional aspects and their easy implementation, self-report data bears the risk of inaccuracy due to recall bias, social desirability and difficulties in introspective ability. Secondly, the study population predominantly consisted of morbidly obese patients, a condition that frequently entails a considerable number of comorbidities that in turn might act as confounding variables. Third, since we chose a naturalistic study design that usually does not provide a control group, therefore no healthy normal weight subjects were included. The naturalistic design can either be seen as a limitation or a strength. The low degree of standardization in return favors a higher ecological validity. Compared with randomized controlled trials (RCTs) our investigations allow a more comprehensive picture which fits the biopsychosocial approach that is widely acknowledged in the field of psychosomatic medicine. However, it is important to note that no causality can be drawn from our findings since the influence of confounding and interaction effects cannot be entirely eliminated. Lastly, it cannot be excluded that the employed immunoassays recognized certain degradation products of the targeted peptides next to their biologically active forms. Especially with regard to PYY, C-terminal truncation of PYY_3__–__36_ to PYY_3__–__34_ entails the loss of its bioactivity in regulating energy homeostasis (Lafferty et al., 2018). Nonetheless, in the event of an upregulation as observed in PP associated with anxiety, we would expect increased levels of both active as well as inactive forms. Besides, psychometric assessment tools for anxiety and depression used in the present study evaluate symptom severity during the past two weeks while PSQ-20 assesses the patients general experience of stress. As we did not aim to examine acute emotional responses the exact determination of biologically active forms would not appear to be of utmost importance.

Taken together, current data support the concept of the gut-brain axis as bidirectional interplay of peripheral and central signals in the regulation of behavioral patterns essential for survival. In this case the role of the gut-derived PP in the regulation of anxiety is particularly noteworthy, although other factors are very likely to affect this association as well. Peptides of the NPY family are involved in multiple physiological pathways and thus being influenced by many different variables. Our findings emphasize especially age and conceivably sex hormones as distinct contributing factors. Further research employing normal-weight controls as well as the consideration of hormonal status or menstrual cycle phase are required to better understand the role of NPY, PYY and PP in the pathophysiology of obesity and the emotional response to stress.

## Data Availability Statement

The raw data supporting the conclusions of this article will be made available by the authors, without undue reservation, to any qualified researcher.

## Ethics Statement

The studies involving human participants were reviewed and approved by the Ethikkommission – Charité – Universitätsmedizin Berlin. The patients/participants provided their written informed consent to participate in this study.

## Author Contributions

EWö, EWe, and SS collected the samples. SS analyzed the data and wrote the first draft of the manuscript. TH and AS designed the study and gave critical input throughout the work. AS analyzed the data. All authors finalized and agreed on the final version of the manuscript.

## Conflict of Interest

The authors declare that the research was conducted in the absence of any commercial or financial relationships that could be construed as a potential conflict of interest.
